# Cost-effectiveness of the mobile application TCApp combined with face-to-face CBT treatment compared to face-to-face CBT treatment alone for patients with an eating disorder: study protocol of a multi-centre randomised controlled trial

**DOI:** 10.1186/s12888-018-1664-4

**Published:** 2018-05-02

**Authors:** Dimitra Anastasiadou, Francisco Lupiañez-Villanueva, Clara Faulí, Jordina Arcal Cunillera, Eduardo Serrano-Troncoso

**Affiliations:** 1 Department of Information and Communication Sciences, Universitat Oberta de Catalunya, Barcelona, Spain; 2 Open Evidence Research Group, Universitat Oberta de Catalunya, Barcelona, Spain; 3 HealthApp, Barcelona, Spain; 4 Child and Adolescent Psychiatry and Psychology Department, Hospital Sant Joan de Déu of Barcelona, Passeig Sant Joan de Déu, 2, 08950 Esplugues, Spain; 5 Children and Adolescent Mental Health Research Group, Institut de Recerca Sant Joan de Déu, Santa Rosa 39-57, 08950 Esplugues de Llobregat, Spain

**Keywords:** Eating disorders, mHealth, Treatment, Cost-effectiveness

## Abstract

**Background:**

The clinical utility of the existing apps for people with eating disorders (EDs) is not clear. The TCApp has been specifically developed for people with EDs, is based on the principles of Cognitive Behavioural Treatment (CBT) and allows a bidirectional link between the patient and the therapist. The objectives of the study are, first, to assess the clinical efficacy of a combined intervention for Eating Disorders (EDs) that includes an online intervention through the TCApp plus standard face-to-face CBT in comparison to standard face-to-face CBT alone, and second, to examine the cost-effectiveness of the TCApp and identify potential predicting, moderating and mediating variables that promote or hinder the implementation of the TCApp in ED units in Spain.

**Methods:**

The study methodology is that of a randomised controlled trial combining qualitative and quantitative methods, with a 6-month follow-up. Approximately 250 patients over 12 years old with a diagnosis of an ED from several ED units in Spain will be randomised to one of two different conditions. Participants, their caregivers, healthcare professionals and technical staff involved in the development and maintenance of the application will be assessed at baseline (T0), post-intervention (T1) and at 6 months follow-up (T2). Primary outcome measures will include ED symptomatology while secondary measures will include general psychopathology and quality of life for patients, quality of life and caregiving experience for family caregivers and adoption-related variables for all participants involved, such as perceived usability, user’s satisfaction and technology acceptance. For the cost-effectiveness analysis, we will assess quality-adjusted life years (QALYs); total societal cost will be estimated using costs to patients and the health plan, and other related costs.

**Discussion:**

The study will provide an important advance in the treatment of EDs; in the long term, it is expected to improve the quality of patient care and the treatment efficacy and to reduce waiting lists as well as direct and indirect costs associated with the treatment of EDs in Spain.

**Trial registration:**

ClinicalTrials.gov: NCT03197519; registration date: June 23, 2017.

## Background

In the last three decades, Eating Disorders (EDs) have become a particularly relevant public health concern due to their growing incidence, the severity of the associated physical and psychiatric comorbidities [1], the high suicide and mortality rates [2] and the resistance of patients to treatment [3], among other reasons. In addition, they constitute today the third most prevalent chronic illness among the adolescent female population in developed and westernized societies and a public health priority around the world [4, 5]. Specifically in Spain, 75,000 adolescents are affected by one of the different manifestations of the disorder, among which 90% are female [6].

Although we do not have specific data related to the economic burden of treatment in Spain, various Cost Of Illness (COI) studies conducted in other European countries indicate that the direct costs for diagnosis and treatment of patients (medical expenditures, nonmedical costs, out-of-pocket expenses of the patient and his/her family), together with the indirect costs (loss of productivity due to sickness, reduced productivity or death) imply a high economic impact [7] and a significant loss of quality of life for people affected [8, 9] and their families [10]. However, according to the systematic review conducted by Stuhldreher et al. (2012), COI studies are sparse, the costs are often underestimated, the majority target only outpatient settings, and due to their heterogeneous nature, it is impossible to carry out comparisons between studies.

The widespread use of new technologies offers a promising and innovative way to improve the quality of care for patients with an ED and to reduce its economic burden. Systematic reviews of the literature demonstrate the effectiveness of eHealth as a complementary therapeutic tool in the treatment of EDs [11–16]. For example, Internet-based interventions have proved their efficacy in reducing ED-related symptoms in a number of Randomised Controlled Trials (RCTs) [17–19]. However, to date, there is a complete absence of RCTs evaluating the effectiveness of mHealth interventions for EDs and existing studies only offer preliminary results about acceptability and usability of such tools [20–22].

In particular, mHealth interventions for EDs may help patients to increase their treatment adherence and to better deal with feelings of stigma associated with face-to-face psychological treatment. Additionally, considering the shortage of ED specialists and the long waiting lists in mental health outpatient settings, mHealth interventions may offer improved accessibility and availability of treatment, at lower costs [20].

In addition, it is worth noting that traditional Cognitive Behavioural Therapy (CBT) is considered the “gold standard” treatment for EDs. CBT uses real-time self-monitoring of patients’ eating habits and behaviours to help them gain a clearer picture of their problem and understand what triggers their behaviour and what are the consequences of it. Self-monitoring is the key driver of behavioural change as it helps patients intervene in the moment [12]. By doing so, all the relevant information regarding the eating problem is entered in real time and can be recovered repeatedly when the therapists or the patients want. Although the efficacy of online CBT interventions as alternatives to face-to-face treatment for patients with EDs is rather controversial [13, 23], such interventions may have a lot of advantages when used as therapeutic tools. Within this context, smartphone apps can offer an attractive and personalised treatment option and can reach a wider range of patients with low motivation for change or others who desire anonymity. Self-monitoring could be enhanced through smartphone-based interventions, as data entry is simpler than traditional pen-and-paper methods. In addition, reminders and motivational messages as well as bidirectional interactions between patient and therapist can improve adherence to treatment [24].

Nevertheless, while growth in mHealth interventions has been rapid, advances in the existing evaluation frameworks have not been seen [25–27]. As a result, a huge number of health apps are widely available to the general healthcare public without possessing best-practice guidelines and certifications or a standardised validation process to assess their long-term cost benefits [28].

The TCApp^1^ represents a tool for connecting patients and therapists in the time between medical consultations. It is currently available on Google Play and Apple Store markets, there are more than 412 patients who are currently using it and has been developed in collaboration with different public and private mental health institutions in the Barcelona area (Althaia, Hospital de Sant Rafael, CST, ITA and Hospital Sant Joan de Déu). The TCApp was designed from the very beginning with therapists’ and patients’ needs and interests in mind. By using the TCApp, patients and therapists are in continuous contact, allowing for a quicker reaction from the therapist according to the patients’ needs.

Through the TCApp, patients can record their thoughts, actions, emotions and whatever the therapists consider relevant for the therapy, since the app can be customized according to the therapy requirements of each specific patient. It involves algorithms based on artificial intelligence that can generate alarms when strategic words (i.e. suicide, death, etc.) are written. It also introduces technologies to allow real-time online contact with therapists and gamification aesthetics with prizes, rewards and reminders to improve patients’ engagement.

The back-office tool for therapists is a web-based platform where therapists can see in real time what their patients have registered (i.e. generation of graphs in a period of time to see parameter comparison and patient evolution) and they can interact in real time with them, using PUSH notifications. The tool is integrated in Azure server in order to ensure accordance with the most restrictive data protection laws and it is prepared for the integration in local management systems of hospitals and clinics. Lastly, there is currently no application available to provide the same services and benefits as the TCApp as most of the available applications contain self-help functionalities, rather than allowing for a bidirectional link between the patient and the therapist.

The purpose of this trial is to conduct a multicentre, randomized controlled trial with 250 patients diagnosed with an ED. In this experiment, the patients from the experimental group will test a mHealth application (TCApp developed by HealthApp) and then, a clinical efficacy analysis and economic evaluations will be performed. To do this, we have set the following three specific objectives:

- To evaluate the clinical efficacy of an intensive intervention that includes both standard face-to-face Cognitive Behavioural Treatment (CBT) (treatment as usual, TAU) plus mHealth intervention using the TCApp, versus the TAU alone.
- To carry out an economic evaluation of the new mHealth intervention and identify factors that promote or hinder the implementation of the TCApp in mental health settings in Spain.
- To analyse the adoption processes of this type of applications by patients and health professionals and identify the determinants of mHealth adoption.

The implementation of the intensive intervention program (TAU + TCApp) would result in a more significant improvement of the ED symptoms compared to the TAU control group. More specifically:

- The application of the intensive mHealth intervention would lead to significantly greater change scores (difference between T0 and T1, which will be also maintained at follow-up, T2) in the primary outcome variable of ED psychopathology, compared to the control group.
- The mHealth intervention would lead to significantly greater change scores (difference between T0 and T1, which will be also maintained at follow-up, T2) in patients’ secondary outcome variables: a) depression symptoms, b) anxiety symptoms, c) readiness to recover, d) suicidal risk, and e) quality of life, compared to the control group.
- Similarly, intensive intervention would result in greater change scores (difference between T0 and T1, which will be also maintained at follow-up, T2) in caregivers’ variables: a) quality of life and b) caregiver burden.

## Methods

### Design

We will follow a mixed-methods approach, combining quantitative and qualitative methods, through a multi-centre RCT with two parallel groups (an intensive intervention group with TAU and TCApp and a TAU control condition) with a 1:1 allocation.

### Sample

The total sample will be approximately 250 patients with a diagnosis of EDs who are currently under treatment, from different public and private mental health services in Spain (Parc Taulí Hospital, Sant Rafael Hospital, Servei de Salut de les Illes Balears, Sant Joan de Déu Hospital, Niño Jesús University Children’s Hospital, San Carlos Clinic Hospital, Dexeus University Hospital of the Quirónsalud group and Eating Disorders Institute ITA).

All patients will receive a standard CBT treatment, counting with the support of a multidisciplinary team of the different ED units (psychiatry, psychology, nutrition, nursing).

#### Inclusion criteria

- Diagnosis of an Eating or Feeding Disorder, based on: a) the Schedule for Affective Disorders and Schizophrenia-Present and Lifetime version (K-SADS-S-PL) (DSM-V criteria) [29] for minor patients or b) the Structural Clinical Interview for DSM-IV (SCID-1) [30] for adult patients. The diagnosis should be one of the following types: Anorexia Nervosa (AN); Bulimia nervosa (BN); Binge Eating Disorder (BED); Other Specified Feeding or Eating Disorder: Atypical Anorexia Nervosa, Bulimia nervosa (of low frequency and/or limited duration), Binge-eating disorder (of low frequency and/or limited duration), purging disorder, night eating syndrome.
- Treatment regimen: Day Hospital or Outpatient treatment, regardless of the illness duration or the severity of the disorder
- Treatment received by ED unit of reference: Standard Cognitive Behavioural Therapy
- Understanding of Spanish, Catalan or English language, depending on the language option chosen by the participant for the TCApp
- Minimal digital skills and availability of proper mobile phone for patients

#### Exclusion criteria

- Age less than 12 years
- Treatment regimen: Inpatient treatment
- Diagnosis of psychosis
- Intellectual disability
- Have a mobile phone with a Windows Phone operating system

### Procedure and randomization

First, all material with information related to the study (research protocol, informed consent, patient information sheet, Data Collection Logbook, safety- and privacy-related issues concerning the TCApp) have been submitted for approval to each one of the Ethical Committees of the participating hospitals. It should be mentioned that the approval of the Ethical Committee of the University leading the study (Open University of Catalonia, UOC) was obtained on February 21st, 2017.

Participants will be recruited after previous recommendation by one of the ED specialists working at each centre. Specialists will do a preliminary screening following the inclusion and exclusion criteria in order to identify potential candidates for the study. Interested individuals will be able to confirm their participation by notifying the ED specialist who will be responsible for their treatment. Then, an information letter and an informed consent form will be delivered to them.

After completing and signing these documents (for underage patients, their parents will be required to sign the informed consent and for patients aged more than 18 years, they will have to sign informed consent themselves), initial clinical interviews will be conducted by psychologists or other collaborators working in the ED unit. All interviewers will be previously trained in administering the K-SADS-PL or SCID interview, depending on the participant’s age. The objective of these interviews is: a) to determine whether participants are definitely eligible for the study following the inclusion criteria, b) to establish the diagnosis for each patient and c) to evaluate them for possible comorbidities. At this time, sociodemographic and clinical data of each patient will also be collected through a brief interview. Then, those who meet the inclusion criteria will be invited to complete the baseline questionnaires for the study. During this baseline evaluation (T0), questionnaires will be administered to patients, their informal caregivers and the ED specialist responsible for the online monitoring of each patient. In addition, telephone interviews will be conducted with the technical staff and the ED specialists.

After completion of the baseline questionnaires, participants will be randomized to one of the two study conditions (experimental or control group). Randomization will be carried out by an independent researcher in blocks of 10 participants within each ED unit (50% of patients from each block will be assigned to the experimental group and the other 50% to the control group), using a random allocation program. Allocation concealment will be also ensured.

After this, patients will be notified about the group they belong to during their next visit to the ED unit. At this time, patients from the experimental group will be given oral and written instructions about how to download and use the TCApp. ED specialists responsible for the online monitoring will be also taught the basic principles for using the application. In turn, patients from the TAU control group will be told that access to the TCApp will be offered to them after a waiting period of 6 months (T2 evaluation completed).

Then, each group of patients will receive the treatment that corresponds to them during a period of 12 weeks. At the end of the 12-week treatment, patients from the experimental group will stop using the TCApp and the evaluation post-treatment (T1, 12 weeks later) will be carried out, including: a) a brief clinical interview (patients), b) questionnaires (patients, informal caregivers, ED specialists); c) telephone interviews (technical staff, ED specialists) and d) focus groups with ED specialists of each institution who are interested in participating as well as with patients of the experimental group.

In the follow-up evaluation T2, 6 months after the beginning of treatment, patients, their informal caregivers and ED specialists responsible for the online monitoring will complete some questionnaires. In addition, telephone interviews will be conducted with ED specialists and a brief clinical interview will be carried out with patients. At the end of this phase, patients from the control group will be given access to the TCApp after being contacted by the ED specialist who will be responsible for their online monitoring.

**Fig. 1:**
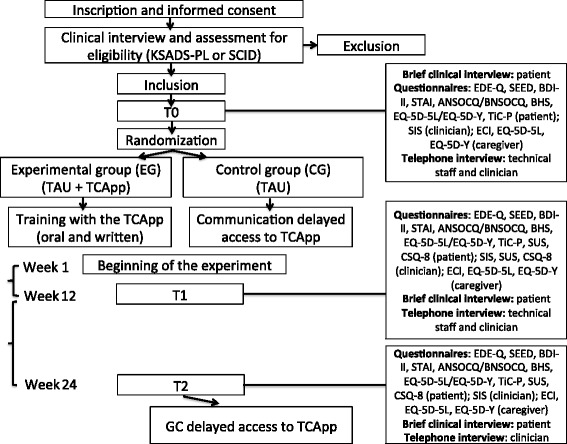
Timeline and procedure

Figure 1 presents the procedure and the timeline of the study and Table 1 provides a detailed description of the methodology with a definition of the study variables and their assessment tools.

**Table 1:** Instruments for measuring clinical efficacy and cost-effectiveness

Dimension	Methodology	Assessment measures	Stakeholder	T0	T1	T2
Clinical efficacy						
Psychopathology	Clinical interview	**Minors** (6-18 years old): Kiddie Schedule for Affective Disorders and Schizophrenia-Present and Lifetime version (K-SADS-PL) (DSM-5) **Adults**: Structured Clinical Interview for DSM Disorders (SCID-I)	Patient	x		
Physical and clinical variables	Brief clinical interview	Comorbidities, medication, illness duration, Body Mass Index, among others	Patient	x	x	x
Eating Disorder psychopathology	Questionnaire	- Eating Disorders Examination Questionnaire (EDE-Q) - Short Evaluation of Eating Disorders (SEED)	Patient	x	x	x
Depression	Questionnaire	Beck Depression Inventory-II (BDI-II)	Patient	x	x	x
Anxiety	Questionnaire	State-Trait Anxiety Inventory (STAI)	Patient	x	x	x
Motivation to change	Questionnaire	**Patients with Anorexia Nervosa**: Anorexia Nervosa Stages of Change Questionnaire (ANSOCQ) **Patients with Bulimia Nervosa or Binge Eating Disorder**: Bulimia Nervosa Stages of Change Questionnaire (BNSOCQ)	Patient	x	x	x
Dropout	Telephone interview	Reasons for dropout	Clinician responsible for the online monitoring		x	x
Risk of suicide	Questionnaire	Beck Hopelessness Scale (BHS) / Suicide Intent Scale (SIS)	Patient / Clinician responsible for the online monitoring	x	x	x
Quality of life	Questionnaire	**12-14 years old**: EQ-5D-Y (children version) **> 14 years old**: EQ-5D-5 L	Patient	x	x	x
Caregiving experience	Questionnaire	Experience of Caregiving Inventory (ECI)	Family caregiver	x	x	x
Quality of life	Questionnaire	EQ-5D-5 L	Family caregiver	x	x	x
Cost-utility and cost-effectiveness analysis						
Cost related to the development and maintenance of the online platform	Telephone interview	Staff involved, number of extra hours x pay rate/hour	Technical staff	x	x	
Costs related to healthcare utilization, medication and school and / or work absenteeism	Questionnaire	iMTA questionnaire for Costs associated with Psychiatric Illness (TiC-P) / iMTA questionnaire for Costs associated with Psychiatric Illness, Parent-Form (TiC-P Children)	Adult patient / family caregiver (referring to minor patient)	x	x	x
Patient’s healthcare utilization	Telephone interview	Number of visits to ED specialists, number of visits to the emergency department, medication consumption and its costs	Clinician responsible for the online monitoring	x	x	x
Cost of implementing the online intervention	Telephone interview	Clinicians involved, number of extra hours for online monitoring x pay rate/hour	Clinician responsible for the online monitoring		x	
Usability and satisfaction	Questionnaire	- Client Satisfaction Questionnaire (CSQ-8) - System Usability Scale (SUS)	ED specialists and patients from the experimental group		x	

### Study conditions

The experimental group will receive the standard treatment based on CBT principles that is offered by the different ED units in Spain, plus an online intervention using the TCApp for a period of 12 weeks. Only one ED specialist will be responsible for the online monitoring of each patient. For the specific purposes of our study, this role has been assigned to the nursing staff for most of the centres.

The TCApp application proposes patients a series of different functionalities, including daily self-records of their thoughts, emotions and actions, a chat with their therapists and motivational exercises. An online platform of the TCApp (BackOffice) is also available for therapists for the online monitoring of each patient. There, therapists have the possibility to follow the patient’s daily self-records, generate personalized reports and graphs and communicate with him/her via chat, based on the information that the patient has provided online.

During these 12 weeks, the patient should use the TCApp at least once a day, completing at least one self-record per day and/or contacting his/her therapist via chat when needed. The therapist responsible for the online monitoring should connect to the online platform and perform the following actions at least once a week: follow the patient’s daily self-records, generate personalized reports or graphs and communicate with him/her via chat. After a 12-week period, patients from the experimental group and their therapists will stop using the TCApp (they will be discharged).

The TAU control group will receive the standard face-to-face CBT, offered by the different ED units in Spain. Patients from the control group will be offered access to the TCApp after a 6-month period.

### Measurements

#### Primary outcome measures (for patients)

*Eating Disorder Examination Questionnaire* (EDE-Q) [31, 32]. The EDE-Q is derived from the Eating Disorder Examination Interview (EDE) (Fairburn & Cooper, 1993) and is commonly used to assess the frequency of core ED behaviors and the attitudinal features related to ED pathology over the past 28 days. Twenty-two of the 36 items assess attitudes related to ED pathology and are divided into four subscales (concerns about weight, shape and eating, and restraint). Items are rated on a 7-point Likert scale ranging from 0 = not 1 day/not at all, to 6 = every day/markedly. The core ED behaviors (remaining 14 items) are assessed in terms of their presence and frequency. A global score of eating psychopathology can be calculated by summing and averaging all of the items. Higher scores indicate higher ED psychopathology. The EDE-Q has demonstrated good reliability and validity both in its original version [33] and in Spanish [32].

*The Short Evaluation of Eating Disorders* (SEED) [8, 34]. The SEED was developed for the assessment of key ED symptoms. It includes 3 items related to AN symptoms (degree of underweight, fear of weigh gain and distortion of body perception) and 3 items related to BN symptoms (amount of binge eating episodes, amount of compensatory behaviors and over concern with body shape and weight). The questionnaire allows the calculation of two severity indices for AN and BN (range from 0 to 3). The construct validity and the criterion-related validity of the SEED yielded positive results and the sensitivity to change of the instrument was also satisfactory [34].

#### Secondary outcome measures

##### For patients

*Beck Depression Inventory* (BDI-II) [35]. The BDI-II is an instrument used for assessing the severity of depression in adult and adolescent patients over a period of 2 weeks. It includes 21 items, which are answered on a 4-point Likert scale ranging from 0 (not at all) to 3 (extreme form of each symptom). The total score is obtained by summing the severity ratings of each depression item. The questionnaire showed strong internal consistency and good test-retest reliability in both English and Spanish languages, among clinical and non-clinical samples [36, 37].

*State-Trait Anxiety Inventory* (STAI) [38]. The STAI is composed of 20 items for assessing trait anxiety and 20 items for state anxiety. Responses are on a 4-point Likert scale, ranging from “Almost never” to “Almost always”. Higher scores indicate higher levels of anxiety. The instrument showed satisfactory reliability with Cronbach’s α ranging from 0.86 to 0.95 and also test-retest reliability coefficients ranged from 0.65 to 0.75 over a 2-month interval [38]. The Spanish validation by Spielberger, Gorshuch and Lushene [39] showed satisfactory reliability for State Anxiety (0.90-0.93) and for Trait Anxiety (0.84-0.87). Test-retest reliability varied 0.73 and 0.86 on STAI-T [40].

*Anorexia Nervosa Stages of Change Questionnaire* (ANSOCQ) [41] and the *Bulimia Nervosa Stages of Change Questionnaire* [42] will be used to measure patients’ readiness to recover behaviours and attitudes related to their eating disorder. In particular, the ANSOCQ is used for AN patients and the BNSOCQ for patients with BN or Binge Eating Disorder. Both questionnaires are composed of 20 items and scores on each item range from 1 to 5, following stages of change model by Prochaska and colleagues (1998) [43], according to which "1" signifies the Precontemplation stage, "2" is for Contemplation, "3" is for Preparation, "4" is for Action and "5" is for Maintenance. Total scores are obtained by summing the individual items and range from 20 to 100. The ANSOCQ showed good internal consistency and one-week test-retest reliability, both in the English and Spanish version [44]. In turn, the validation study of the BNSOCQ, which was conducted in Spain, demonstrated good internal consistency with Cronbach’s alpha of 0.94 and one-week test-retest reliability of 0.93 [42].

*Beck Hopelessness Scale* (BHS) [45]. The BHS is a 20-item questionnaire designed to measure negative attitudes about the future in clinical and research settings. More specifically, it measures three major aspects of hopelessness: feelings about the future, loss of motivation and expectations. The Spanish validation of the instrument showed adequate reliability (Cronbach’s α ranging from 0.82 to 0.84) and moderate construct validity [46].

*EQ-5D-5 L* and *EQ-5D-Y* [47]. The EuroQoL-EQ-5 L is a standardized instrument used as a health outcome measure for economic evaluation studies. It consists of five dimensions: mobility, self-care, usual activities, pain/discomfort and anxiety/depression. Each dimension has 5 levels: no problems, slight problems, moderate problems, severe problems and extreme problems. The questionnaire also includes an EQ Visual Analogue scale (EQ VAS) about how good or bad the individual’s health is today. The EQ-5D-5 L has been validated in 6 countries, including different groups of patients with chronic conditions and a student cohort. The psychometric properties of EQ-5D-5 L were superior to the previous version of the instrument (EQ-5D-3 L) in terms of validity, although reliability remains to be assessed for the EQ-5D-5 L. In addition, the child-friendly EQ-5D version (EQ-5D-Y) [48] will be used for children and adolescents following and same structure as the EQ-5D (descriptive system and EQ VAS).

*iMTA questionnaire for Costs associated with Psychiatric Illness* (TiC-P) [49]. The TiC-P will be used for adult patients only, to evaluate the healthcare utilization due to the psychiatric illness, such as number of patient visits to their general practitioner, medical specialists and paramedics, during the last 3 months. The questionnaire will also assess productivity losses due to absence from work or due to reduced efficiency during paid or unpaid work, as well as medication consumed due to the patient’s psychiatric disorder. The instrument establishes direct medical costs and productivity costs and is widely used among different countries for economic evaluations in mental health. The Spanish version of the questionnaire has been obtained upon permission from the original authors. The feasibility and reliability of the instrument was satisfactory and the construct validity of the questions related to healthcare utilisation and long-term work absenteeism was also good [50].

The *System Usability Scale* (SUS) [51, 52]. The SUS is a 10-item questionnaire with five response options that range from 4 = “Strongly agree” to 0 = “Strongly disagree”. The SUS provides a “quick and dirty” reliable tool for measuring the usability of a range of systems. The participant’s scores for each question are converted to a new number, added together and then multiplied by 2.5 to convert the original scores of 0-40 to 0-100.

*Client Satisfaction Scale* (CSQ-8) [53]. The CSQ-8 is an 8-item questionnaire that is designed to measure client satisfaction with services. Scores are on a 4-point Likert scale and range from 8 to 32, with higher scores indicating greater satisfaction. The instrument showed satisfactory reliability with Cronbach’s α ranging from 0.83 to 0.93 and correlations of the instrument with other measures of general satisfaction ranged from 0.6 to 0.8. The CSQ-8 is also available in Spanish by Martínez and Beiti [54].

##### For clinicians responsible for the online monitoring

*Suicide Intent Scale* (SIS) [55]. The SIS is a questionnaire that measures the severity of the suicide attempt and should be fulfilled by the clinician responsible for the treatment of the patient. The scale consists of 20 questions, which are scaled from 0 to 2 and cover three aspects: 1) objective circumstances surrounding the attempt, 2) perceptions of potential lethality, expectations of rescue, purpose of the attempt, impulsivity and reaction to the attempt and 3) other aspects. Both the English and the Spanish version of the instrument showed satisfactory reliability and validity [55, 56].

The *System Usability Scale* (SUS) [51, 52].

*Client Satisfaction Scale* (CSQ-8) [53].

##### For caregivers

*Experience of Caregiving Inventory* (ECI) [57]. The ECI is composed of 66-items and is designed to measure how a person caring for someone with a serious mental illness appraises his/her experience. It is composed of ten subscales, eight of which measure negative aspects of caregiving (difficult behaviors, negative symptoms, stigma, problems with services, effects on family, need to backup, dependency and loss) and two measure positive aspects (rewarding personal experiences and good aspects of relationship with the patient), which can be grouped into two subscales: the ECI-negative and the ECI-positive. Responses are on a 5-point Likert scale (ranging from 0 = “never” to 4 = “nearly always”). Higher scores indicate a more positive or negative appraisal of the caregiving experience. The ten scales showed satisfactory reliability with Cronbach’s α ranging from 0.74 to 0.91. The internal reliability coefficients of the caregivers were 0.81 for the ECI-positive and 0.92 for the ECI-negative.

*EQ-5D-5 L* [48].

*iMTA questionnaire for Costs associated with Psychiatric Illness, Parent-Form* (TiC-P Children). The Dutch version of the TiC-P Children has been used to measure medical and non-medical costs in children with mental health disorders. The questionnaire was obtained upon permission from the original authors and was translated from Dutch into Spanish by our research group.

#### Qualitative assessment

In order to perform the economic evaluation, all relevant costs due to healthcare utilization (number of visits to ED specialists, number of visits to the emergency department, medication consumption and its costs) as well as other costs related to the implementation of the online intervention with the TCApp for both clinicians responsible for the online treatment and technicians (amount of extra working hours) will be evaluated through a telephone interview with each stakeholder.

### Sample size calculation

The a priori sample size calculation was based on results from previous studies that implemented Internet-based programs in the treatment of EDs [58–60]. A small between-group effect size (Cohen’ s d = 0.40) is expected. The calculation was conducted by the software program G*POWER. The primary analysis will concern the hypothesis that the average level of eating pathology at post-intervention in the control group, based on the EDE-Q scores, will be higher than the average levels of eating pathology in the experimental group. Assuming an alpha of 0.05 and a power of 0.80 (β − 1) in an independent samples one-way t-test study, a minimum of 100 participants would be required per study arm. Allowing for a dropout rate of 25% of study participants from baseline, a total of 250 participants need to be recruited.

### Data management

All data related to the trial, including clinician’s paper notes of the diagnostic interviews (K-SADS, SCID), self-report questionnaires, informed consent forms, and audio recordings of the telephone interviews, will be securely stored in the workplace of our research group located in the UOC. Only authorised researchers directly involved in the study will have access to this information. According to the UOC’s approval report, all obtained data will be kept for a minimum of 5 years. After obtaining the signed informed consent from participants, a unique code will be allocated to each one of them. The file that links participants to their codes and the databases will be also securely stored on a secure server, password-accessible only to the research team.

### Statistical analysis

Both intent-to-treat and completers analyses will be carried out. Intent-to-treat analysis will include every participant who will be randomly allocated to one of the study conditions, that is to say, 250 patients. Whenever possible, we will try to collect follow-up data from participants who have dropped out, in order to keep our dataset as complete as possible. Baseline differences between completers and dropouts will be analysed (Pearson’s chi-square test Student’s t test) using data from the clinical interviews and the baseline questionnaires and possible reasons for dropout will be examined through interviews with the ED specialists (T1, T2).

A participant will be considered a completer if he/she has completed the initial clinical interview as well as T0 and T1 evaluations. For participants from experimental group, to be considered completers they will have to have used the TCApp at least 70% of the time that was initially agreed upon before the start of the experiment (at least once a day during a period of 12 weeks). Only data from completers will be used to determine the treatment effect on the main outcome variable.

All analyses will be carried out using SPSS (Statistical Package for the Social Sciences). First, we will examine the data using descriptive statistics. Between-group analysis will be conducted using Pearson’s chi-square test or Fisher’s exact test for categorical data and Student’s t test or the Mann-Whitney U test for continuous variables, depending on the normality of the distribution. The normality of the distribution of the variable will be assessed using the Kolmogorov-Smirnov test. The effect of the intervention in terms of reduction in the primary and secondary outcome measures over time will be computed by using a mixed model linear regression analysis with time, experimental condition and their interaction as independent variables. Since there may be some heterogeneity in the implementation of the intensive online intervention across the different participating institutions, between-centre heterogeneity will be explored in subgroup analyses.

### Economic evaluation

A cost-effectiveness analysis of the TCapp will be performed, which will compare the effects and costs of the intensive online intervention (TAU + TCapp) with the ones for TAU. The effects will be measured with quality-adjusted life years (QALYs) obtained from the EQ-5D-5 L and EQ-5D-Y questionnaires. Furthermore, the effects regarding the primary outcome variables (EDE-Q, SEED) will also be taken into account. On the other hand, costs will cover the development of the App, the costs of using the product, and the medical costs (visits and medication) for both groups. Moreover, societal costs such as productivity losses and the caregiving burden will also be assessed. Information on costs will be obtained from the iMTA questionnaire and from interviews with the clinicians and technicians. This evaluation will determine the incremental costs and effects of the intensive intervention compared to the control condition , and will produce an incremental cost-effectiveness ratio (ICER). 

## Discussion

In this article  we present the protocol of a study aimed to assess the clinical efficacy of an intensive intervention program using the TCApp. In addition to the assessment of changes in ED pathology and other secondary outcomes, such as anxiety, depression and quality of life of both patients and their caregivers, we will assess the differential cost-effectiveness of an intensive treatment (TCApp + TAU) compared to that of TAU. This is an important strength of this study because to our knowledge, there are only a few apps for mental disorders with supporting evidence from RCTs and none of them was specifically designed for people with EDs or ED professionals [11, 61]. Once the TCApp has proven to be an efficient and cost-effective tool for use in ED units in Spain, the long-term contributions of the current study are as follows: 1) to promote the clinical use of the TCApp in ED units not only in Spain but also on an international level, 2) to improve the quality of patient care using the TCApp as complementary tool alongside face-to-face psychological treatment, while reducing direct and indirect costs associated with the treatment of the illness and 3) to explore the future use of the application in other mental disorders whose treatment is based on CBT, such as depression, addictions and anxiety.

Among the strengths of the study is its large sample size, which is clinically relevant, as it will be recruited from different public and private ED units in Spain. Another strength is the use of face-to-face assessment and a diagnostic interview to establish patient’s clinical diagnosis and confirm his/her final inclusion in the study, according to the inclusion and exclusion criteria. Furthermore, the assessment of the experience of caregivers is another strength worth mentioning.

Regarding the limitations of the study, the short follow-up time as well as the delayed access to the TCApp by the control group, which may influence our results, should be mentioned. Finally, a limitation related to the nature of the study is the fact that it is a multi-centre study that includes several private and public ED units in Spain. To overcome this limitation, when performing the statistical analyses, between-centre heterogeneity will be explored in subgroup analyses.

## Abbreviations

AN: Anorexia Nervosa
ANSOCQ: Anorexia Nervosa Stages of Change Questionnaire
BDI: Beck Depression Inventory
BED: Binge Eating Disorder
BHS: Beck Hopelessness Scale
BN: Bulimia Nervosa
BNSOCQ: Bulimia Nervosa Stages of Change Questionnaire
CBT: Cognitive Behavioural Treatment
COI: Cost Of Illness
CSQ-8: Client Satisfaction Scale
DSM: Diagnostic and Statistical Manual of Mental Disorders
ECI: Experience of Caregiving Inventory
EDE: Eating Disorder Examination Interview
EDE-Q: Eating Disorder Examination Questionnaire
EDs: Eating Disorders
ICER: Incremental Cost-Effectiveness Ratio
K-SADS-S-PL: Schedule for Affective Disorders and Schizophrenia-Present and Lifetime version
QALYS: Quality-Adjusted Life Years
RCT: Randomised Controlled Trial
SCID-1: Structural Clinical Interview for Axis 1
SEED: Short Evaluation of Eating Disorders
SIS: Suicide Intent Scale
SPSS: Statistical Package for the Social Sciences
STAI: State-Trait Anxiety Inventory
SUS: System Usability Scale
TAU: Treatment As Usual
UOC: Open University of Catalonia
VAS: Virtual Analogue Scale

## Footnotes

1
http://www.bcnhealthapp.com

## References

[1] Agras WS (2001). The consequences and costs of the eating disorders. Psychiatr Clin North Am.

[2] Preti A, Rocchi MBL, Sisti D, Camboni MV, Miotto P (2011). A comprehensive meta-analysis of the risk of suicide in eating disorders. Acta Psychiatr Scand.

[3] Hötzel K, von Brachel R, Schlossmacher L, Vocks S, Israël M, Oxman A (2013). Assessing motivation to change in eating disorders: a systematic review. J Eat Disord.

[4] Austin B. The blind spot in the drive for childhood obesity prevention: bringing eating disorders prevention into focus as a public health priority. Am J Public Health. 2011;101:1–5. 10.2105/AJPH.2011.300182. 10.2105/AJPH.2011.300182 21493926

[5] Gandarillas A, Sepulveda AR (2003). Epidemiological surveillance of eating disorders and related behaviors [Vigilancia epidemiológica de los trastornos del comportamiento alimentario y conductas asociadas]. Bol Epidemiológico CAM.

[6] Cervera M. Riesgo y prevención de la anorexia y la bulimia. Madrid: Pirámide; 2005.

[7] Stuhldreher N, Konnopka A, Wild B, Herzog W, Zipfel S, Löwe B (2012). Cost-of-illness studies and cost-effectiveness analyses in eating disorders: a systematic review. Int J Eat Disord.

[8] Kordy H (2005). Counting the COST: a European collaboration on the efficiency of psychotherapeutic treatment of patients with eating disorders. Eur Eat Disord Rev.

[9] Steinhausen H-C (2002). The outcome of anorexia nervosa in the 20th century. Am J Psychiatry.

[10] Anastasiadou D, Medina-Pradas C, Sepulveda AR, Treasure J. A systematic review of family caregiving in eating disorders. Eat Behav. 2014;15:464–77. 10.1016/j.eatbeh.2014.06.001. 10.1016/j.eatbeh.2014.06.001 25064301

[11] Fairburn CG, Rothwell ER. Apps and eating disorders: a systematic clinical appraisal. Int J Eat Disord. 2015;48:1038–46. 10.1002/eat.22398. 10.1002/eat.22398 PMC4737215 25728705

[12] Bauer S, Moessner M (2013). Harnessing the power of technology for the treatment and prevention of eating disorders. Int J Eat Disord..

[13] Loucas CE, Fairburn CG, Whittington C, Pennant ME, Stockton S, Kendall T (2014). E-therapy in the treatment and prevention of eating disorders: a systematic review and meta-analysis. Behav Res Ther.

[14] Aardoom JJ, Dingemans AE, Spinhoven P, Van Furth EF, Beintner I, Jacobi C (2013). Treating eating disorders over the Internet : a systematic review and future research directions. Behav Res Ther.

[15] Juarascio AS, Manasse SM, Goldstein SP, Forman EM, Butryn ML. Review of smartphone applications for the treatment of eating disorders. Eur Eat Disord Rev. 2015;23:1-11. 10.1002/erv.2327. 10.1002/erv.2327 PMC4847127 25303148

[16] Traviss-Turner GD, West RM, Hill AJ. Guided self-help for eating disorders: A systematic review and metaregression. Eur Eat Disord Rev. 2017;25:148–64.10.1002/erv.2507. 10.1002/erv.2507 28276171

[17] Kass AE, Trockel M, Safer DL, Sinton MM, Cunning D, Rizk MT, et al. Internet-based preventive intervention for reducing eating disorder risk: a randomized controlled trial comparing guided with unguided self-help. Behav Res Ther. 2014;631:90–8. 10.1016/j.brat.2014.09.010. 10.1016/j.brat.2014.09.010 PMC4383716 25461783

[18] Hötzel K, Von Brachel R, Schmidt U, Rieger E, Kosfelder J, Hechler T, et al. An internet-based program to enhance motivation to change in females with symptoms of an eating disorder : a randomized controlled trial. Psychol Med. 2014;44:1947–63. 10.1017/S0033291713002481. 10.1017/S0033291713002481 24128818

[19] Sanchez-Ortiz V, Munro C, Stahl D, J H, Startup H, Treasure J, et al. A randomized controlled trial of internet-based cognitive-behavioural therapy for bulimia nervosa or related disorders in a student population. Psychol Med. 2011;41:407–17. 10.1017/S0033291710000711. 10.1017/S0033291710000711 20406523

[20] Juarascio AS, Goldstein SP, Manasse SM, Forman EM, Butryn ML. Perceptions of the feasibility and acceptability of a smartphone application for the treatment of binge eating disorders : qualitative feedback from a user population and clinicians. Int J Med Inform. 2015;84:808–16. 10.1016/j.ijmedinf.2015.06.004. 10.1016/j.ijmedinf.2015.06.004 PMC4860812 26113461

[21] Nitsch M, Dimopoulos CN, Flaschberger E, Saffran K, Kruger F, Garlock L, et al. A Guided Online and Mobile Self-Help Program for Individuals With Eating Disorders: An Iterative Engagement and Usability Study. J Med Internet Res. 2016;18:1–11. 10.2196/jmir.4972. 10.2196/jmir.4972 PMC4726867 26753539

[22] Tregarthen JP, Lock J, Darcy AM. Development of a smartphone application for eating disorder self-monitoring. Int J Eat Disord. 2015;48:972-82. 10.1002/eat.22386. 10.1002/eat.22386 26213130

[23] Dölemeyer R, Tietjen A, Kersting A, Wagner B, Hoek H, Van Hoeken D (2013). Internet-based interventions for eating disorders in adults: a systematic review. BMC Psychiatry.

[24] Fairburn CG, Patel V (2014). The global dissemination of psychological treatments: a road map for research and practice. Am J Psychiatry.

[25] Schweitzer J, Synowiec C (2012). The economics of eHealth and mHealth. J Health Commun.

[26] World Health Organization. mHealth: New horizons for health through mobile technologies. Geneva; 2011. http://www.who.int/goe/publications/goe_mhealth_web.pdf. Accessed 17 July 2017.

[27] Kazanjian A, Green CJ (2002). Beyond effectiveness: the evaluation of information systems using a comprehensive health technology assessment framework. Comput Biol Med.

[28] Leigh S, Flatt S. App-based psychological interventions : friend or foe ? Evid Based Ment Heal. 2015;18:25–8. 10.1136/eb-2015-102203. 10.1136/eb-2015-102203 PMC11234577 26459466

[29] Kaufam J, Birmaher B, Brent D, Rao U, Flunn C, Moreci P, et al. Schedule for affective disorders and schizophrenia for school-age children-present and lifetime version (K-SADS-PL): initial reliability and validity data. J Am Acad Child Adolesc Psychiatry. 1997;36:980–8. 10.1097/00004583-199707000-00021. 10.1097/00004583-199707000-00021 9204677

[30] First M, Spitzer R, Gibbon M, Williams J (2002). Structured clinical interview for DSM-IV-TR Axis I disorders, research version, patient edition. (SCID-I/P).

[31] Fairburn C, Beglin S. Assessment of eating disorder psychopathology: interview or self-report questionnaire? Int J Eat Disord. 1994;16:363–70. 10.1016/j.brat.2003.07.008. 7866415

[32] Peláez-Fernández MA, Javier Labrador F, Raich RM. Validation of eating disorder examination questionnaire (EDE-Q)--Spanish version--for screening eating disorders. Span J Psychol. 2012;15:817–24. 10.5209/rev_sjop.2012.v15.n2.38893 22774455

[33] Berg KC, Peterson CB, Frazier P, Crow SJ (2012). Psychometric evaluation of the eating disorder examination and eating disorder examination-questionnaire: a systematic review of the literature. Int J Eat Disord..

[34] Bauer S, Winn S, Schmidt U, Kordy H (2005). Construction, scoring and validation of the short evaluation of eating disorders (SEED). Eur Eat Disord Rev.

[35] Beck AT, Steer RA, Ball R, Ranieri WF (1996). Comparison of Beck depression inventories-IA and-II in psychiatric outpatients. J Pers Assess.

[36] Richter P, Werner J, Heerlein A, Kraus A, Sauer H. On the validity of the Beck Depression Inventory. A review. Psychopathology. 1998;31:160–8. 10.1159/000066239. 10.1159/000066239 9636945

[37] Wiebe JS, Penley JA (2005). A psychometric comparison of the Beck depression inventory-II in English and Spanish. Psychol Assess.

[38] Spielberger C, Gorsuch R, Lushene R, Vagg P, Jacobs GA. Manual for the State-Trait Anxiety Inventory. Palo Alto: Consulting Psychologists Press; 1983.

[39] Spielberger C, Gorsuch R, Lushene R (1982). STAI. Cuestionario de Ansiedad Estado/Rasgo.

[40] Universidad Complutense de Madrid. Proyecto de Apoyo a la Evaluación Psicológica Clínica. Inventario de Ansiedad Estado-Rasgo. State-Trait Anxiety Inventory. 2006. https://webs.ucm.es/info/psclinic/evaluacion/ProyectoApoyoEPC2006/INSTRUMENTOSEVALUACION/TRASTORNOSDEANSIEDAD/EVALUACIONGENERALDELOSTRASTORNOSDEANSIEDAD/ESCALADEANSIEDADESTADORASGO(STAI)/STAI_P.pdf. Accessed 3 Aug 2017.

[41] Rieger E, Touyz SW, Beumont PJV (2002). The anorexia nervosa stages of change questionnaire (ANSOCQ): information regarding its psychometric properties. Int J Eat Disord..

[42] Martinez E, Castro J, Bigorra A, Morer A, Calvo R, Vila M (2007). Assessing motivation to change in bulimia nervosa: the bulimia nervosa stages of change questionnaire. Eur Eat Disord Rev.

[43] Prochaska JO, Velicer WF, DiClemente CC, Fava J. Measuring processes of change: applications to the cessation of smoking. J Consult Clin Psychol. 1988;56:520–8. 10.1037//0022-006x.56.4.520 3198809

[44] Serrano E, Castro J, Ametller L, Martínez E, Toro J (2004). Validity of a measure of readiness to recover in Spanish adolescent patients with anorexia nervosa. Psychol Psychother.

[45] Beck AT, Weissman A, Lester D, Trexler L. The measurement of pessimism: the hopelessness scale. J Consult Clin Psychol. 1974;42:861–5. 10.1037/h0037562 4436473

[46] Aguilar E, Hidalgo M, Cano R, López J, Campillo M, Hernández J (1995). Estudio prospectivo de la desesperanza en pacientes psicóticos de inicio: características psicométricas de la escala de desesperanza de Beck en este grupo. An Psiquiatr.

[47] EuroQol Group. EuroQol--a new facility for the measurement of health-related quality of life. Health Policy. 1990;16:199–208. 10.1016/0168-8510(90)90421-9 10109801

[48] EuroQol Group. EQ-5D. https://www.euroqol.org. Accessed 17 July 2017.

[49] Hakkaart-van Roijen L, Donker M, Tiemens B (2002). Trimbos/iMTA questionnaire for costs associated with psychiatric illness (TiC-P): Handleiding.

[50] Bouwmans C, De Jong K, Timman R, Zijlstra-Vlasveld M, Van der Feltz-Cornelis C, Tan Swan S (2013). Feasibility, reliability and validity of a questionnaire on healthcare consumption and productivity loss in patients with a psychiatric disorder (TiC-P). BMC Health Serv Res.

[51] Brooke J. ‘SUS-A quick and dirty usability scale.’ Usability evaluation in industry. London, UK: Taylor & Francis Ltd; 1996.

[52] Brooke J (2013). SUS: a retrospective. J Usability Stud.

[53] Larsen DL, Attkisson CC, Hargreaves WA, Nguyen TD (1979). Assessment of client/patient satisfaction: development of a general scale. Eval Program Plann.

[54] Martínez Azumendi O, Beitia Fernández M. Satisfacción, cumplimiento de expectativas y valoración de la ayuda percibida, en primeras consultas en un Centro de Salud Mental. Psiquis (Mexico). 2000;21:9–26.

[55] Beck A, Schuyler D, Herman I. Development of suicidal intent scales. In: Beck A, Resnik H, Lettieri D, editors. The prediction of suicide. Bowie: Charles Press; 1974. p. 45–56.

[56] Bobes García J, Paz G-Portilla M, Fernández MTB, Martínez PAS, M Bousoño G (2002). Banco de instrumentos básicos para la práctica de la psiquiatría clínica.

[57] Szmukler GI, Burgess P, Herrman H, Benson A, Colusa S, Bloch S (1996). Caring for relatives with serious mental illness: the development of the experience of caregiving inventory. Soc Psychiatry Psychiatr Epidemiol.

[58] Aardoom JJ, Dingemans AE, Spinhoven P, Roijen LH, Van Furth EF (2013). An internet-based intervention for eating disorders consisting of automated computer-tailored feedback with or without supplemented frequent or infrequent support from a coach: study protocol for a randomized controlled trial. Trials.

[59] Hötzel K, von Brachel R, Schmidt U, Rieger E, Kosfelder J, Hechler T (2014). An internet-based program to enhance motivation to change in females with symptoms of an eating disorder: a randomized controlled trial. Psychol Med.

[60] Ruwaard J, Lange A, Broeksteeg J, Renteria-Agirre A, Schrieken B, Dolan CV (2013). Online cognitive-Behavioural treatment of bulimic symptoms: a randomized controlled trial. Clin Psychol Psychother.

[61] Donker T, Petrie K, Proudfoot J, Clarke J, Birch M-R, Christensen H (2013). Smartphones for smarter delivery of mental health programs: a systematic review. J Med Internet Res.

